# CAA proteomics meta-analysis reveals novel targets, key players, and the effects of sex, APOE, and brain region in humans

**DOI:** 10.1007/s00401-025-02886-3

**Published:** 2025-04-29

**Authors:** Curran Varma, Cynthia A. Lemere

**Affiliations:** 1 Department of Neurology, Ann Romney Center for Neurologic Diseases, Brigham and Women’s Hospital, Boston, MA 02115 USA; 2 Department of Neurology, Harvard Medical School, Boston, MA 02115 USA

Cerebral amyloid angiopathy (CAA) is a vascular pathology characterized by beta-amyloid (Aβ) deposition in the cerebrovasculature. Though it can form independently of Alzheimer’s disease (AD), CAA is found in nearly all cases of AD and is associated with cognitive decline and intracerebral hemorrhages. Additionally, it is believed to be responsible for amyloid-related imaging abnormalities during Aβ immunotherapy. While CAA type 1 affects cortical capillaries in addition to larger vessels, type 2 excludes capillaries and prefers larger vessels, like the leptomeninges. To investigate the pathophysiology, recent studies [1–4, 7] have selectively extracted CAA vessels from human brains and performed proteomics, which has uncovered novel targets and improved our understanding of vascular amyloid pathology. However, only 340 proteins are common between these datasets. To retain a large overlapping protein set, this meta-analysis included two of these studies [4, 7] to identify consistencies in the CAA proteomic signature, discover new hits, and take advantage of increased statistical power to explore unanswered questions, such as the effects of sex, brain region, and APOE. 

While Zellner and colleagues [7] had samples from 12 CAA, 13 AD, and 12 control cases, Leitner and colleagues [4] obtained CAA(+) and CAA(-) vessels from the same brain for 6 AD cases in addition to 10 controls. Taken together, this study included a total of 59 cases: 18 CAA, 19 AD, and 22 controls (Fig. 1a). To harmonize datasets and control for differences in methodology, Z-scores were calculated relative to controls within each study [5] and protein data was aligned and merged using protein IDs (Supplementary Table 1). Across studies, 1,677 proteins overlapped and were included in this meta-analysis (Fig. 1a). Comparing the CAA and AD groups in the Zellner study yielded 135 differentially expressed proteins (DEPs) with the top hit being OLFML3 (Fig. 1b). In the Leitner study, paired t-tests between CAA(+) and CAA(-) vessels in AD brains resulted in 363 DEPs with APOE as the top hit (Fig. 1c). Next, the Mann–Whitney U test was performed on the merged datasets and the meta-analysis yielded 107 DEPs, 17 of which increased in CAA and remained significant after Benjamini-Hochberg (B.H.) multiple testing correction (Fig. 1d): PTN, APOE, VTN, OLFML3, APCS, GPC1, CLU, SERPINE2, HTRA1, NRXN1, C3, C4A, FBLN7, MFGE8, SPON1, TGFBI, and TIMP3. The STRING database was used to visualize the interaction network between the top hits and demonstrate APOE, VTN, CLU, and APCS to be central to the network (Fig. 1e). Notably, the meta-analysis yielded 25 novel significant hits (bolded) not found in the Zellner or Leitner studies and removed 391 previously significant proteins across both studies (Fig. 1f). Effect sizes and confidence intervals of the differences in protein levels are provided in Supplementary Table 1 to allow interpretation of the biological effects.

**Fig. 1:**
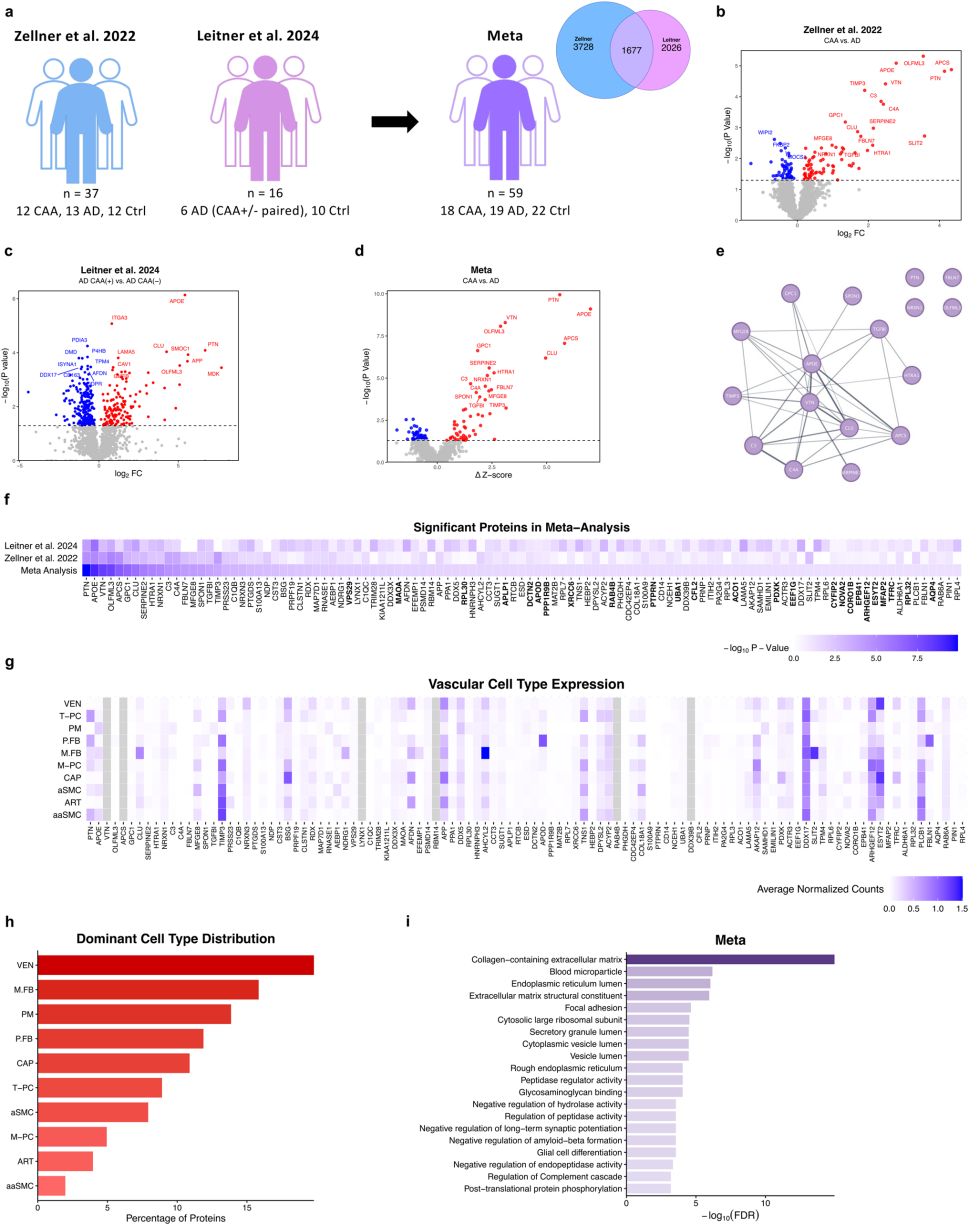
**a** 37 samples from the Zellner et al. study and 16 from the Leitner et al. study were used for this meta-analysis for a total 59 cases, including CAA, AD, and controls. Between proteins sets, 1,677 proteins overlapped and were retained. **b** Volcano plot of DEPs from the Zellner study showing -log_10_ P value and log_2_ FC. Dotted line indicates significance threshold (P < 0.05). Red indicates an increase in CAA compared to AD; blue indicates a decrease. **c** Volcano plot of DEPs from the Leitner study. **d** Volcano plot of meta-analysis DEPs. **e** STRING network of meta hits that passed B.H. correction. **f** Heatmap showing the -log10 P value for all meta hits compared to the original studies. Bolded proteins indicate new hits. **g** Heatmap showing expression levels of meta hits in vascular cell types. **h** Bar plot showing each cell type and the percentage of proteins for which it has the highest (dominant) expression. **i** Bar plot of top 20 pathways for meta hits

Data was extracted from a single-cell vascular atlas of AD brains [6] to identify the cell and vessel types in which the meta-analysis hits could be found as well as their relative expression levels (Fig. 1g). Of the specific cell types, meningeal fibroblasts contributed the most to the CAA proteomic response, followed by perivascular macrophages (Fig. 1h). Finally, “Collagen-containing extracellular matrix” was found to be the top pathway of the meta hits with others involving long-term synaptic potentiation, Aβ formation, glial cell differentiation, and the complement cascade (Fig. 1i).

Upon dividing CAA samples into groups based on APOE genotype, a clear upregulation of the vascular proteome was observed in 3/3 carriers with a notable downregulation in 4/4 carriers, potentially indicating reduced capacity for resilience (Fig. 2a). Similarly, the density plot in Fig. 2b illustrates the negative delta with the 4/4 group relative to 3/3 (Fig. 2b). Further analysis revealed 9 proteins to be significantly different between the two groups, none of which passed B.H. correction, with SERPINA3 being the only to increase in 4/4 carriers (Fig. 2c). There was a striking degree of sex-differences within CAA with 273 DEPs, though none passed B.H. correction. (Fig. 2d). The top hit was TBCD, which was higher in males. DEPs higher in females were heavily involved in the extracellular matrix and cytoskeletal structures, which was similarly found in males with the addition of some relating to synapses (Fig. 2e).

**Fig. 2:**
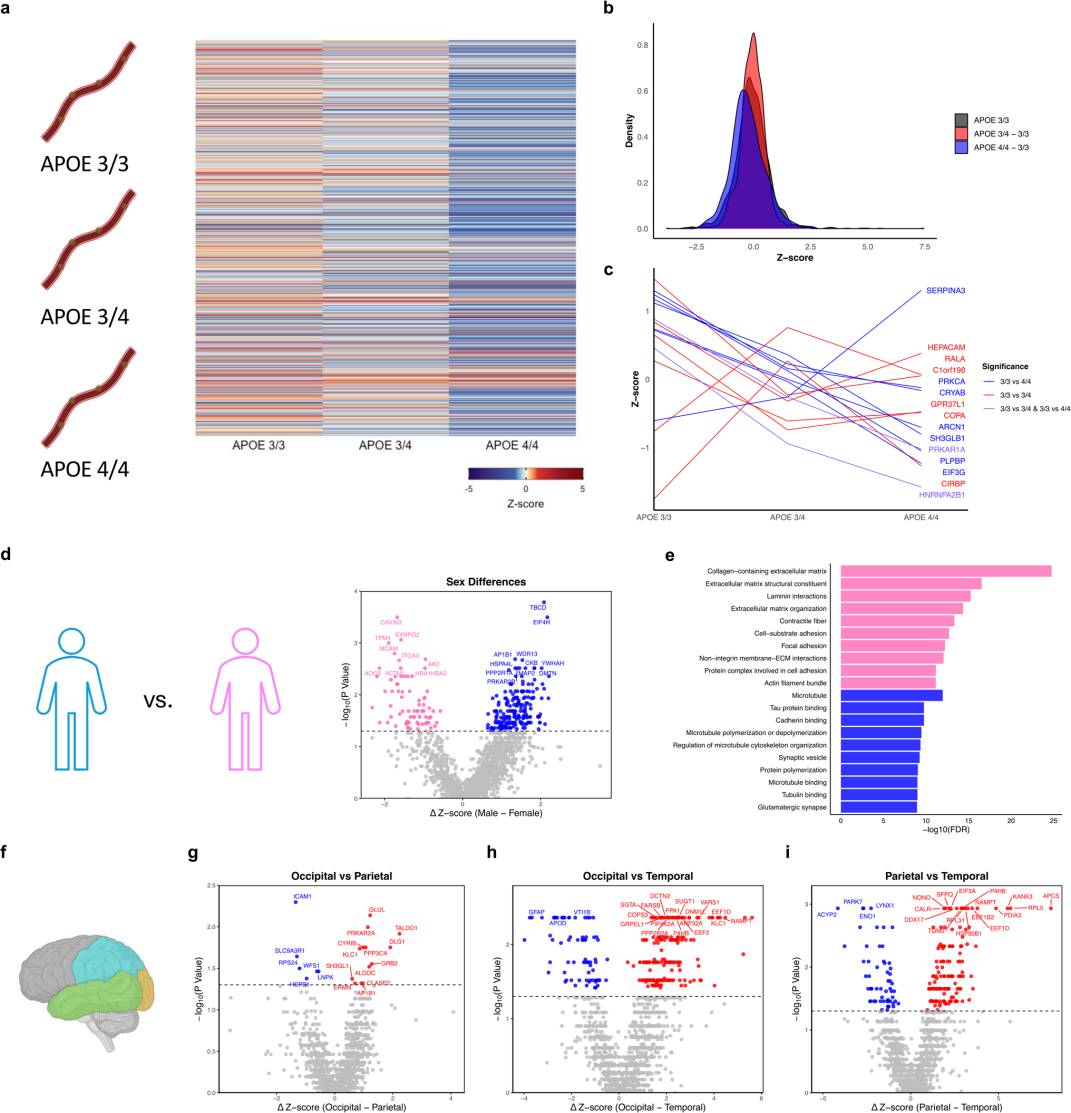
**a** APOE genotypes were compared (left) and the heatmap (right) shows the average expression levels for all proteins in each group. **b** Distribution of Z-scores in the APOE 3/3 group overlayed with subtracted differences compared to the 3/4 and 4/4 groups. **c** Z-scores of significant proteins from Mann––Whitney U tests between APOE groups **d** Sex differences were explored (left) and the volcano plot (right) shows DEPs higher in males (blue) or females (pink). (**e**) Bar plot showing top 10 pathways for DEPs higher in males (blue) or females (pink). **f** Differences between the occipital, parietal, and temporal cortex were assessed. **g** Volcano plot of DEPs higher in occipital cortex (red) or parietal (blue). **h** Volcano plot of DEPs higher in occipital cortex (red) or temporal (blue). **i** Volcano plot of DEPs higher in parietal cortex (red) or temporal (blue)

Numerous differences were found when comparing the CAA proteomes of the occipital, parietal, and temporal cortices (Fig. 2e, f, g, h). For example, 260 DEPs were identified in the occipital vs. temporal analysis (Fig. 2h) and 191 in the parietal vs. temporal analysis with 20 trending after B.H. correction (Fig. 2i). Meanwhile, there were only 19 DEPs in the parietal vs. occipital analysis (Fig. 2g), indicating relatively greater similarity between the regions.

In summary, CAA pathology substantially influences the vascular proteome, and both consistent and novel targets have been identified in this meta-analysis. Additionally, exploring differences with APOE, sex, and brain region revealed large effects. However, this study is not without limitations and would be improved by an increased sample size, which would further power subgroup analyses. Therefore, these results should be considered hypothesis-generating and be validated through larger cohorts. Future studies should investigate the implications of these findings for disease progression and treatment in addition to determining whether any of the top meta hits can serve as or correlate with peripheral fluid biomarkers and how factors like age, disease severity, and post-mortem interval affect CAA proteomic signatures.

## Supplementary Information

Below is the link to the electronic supplementary material.

Supplementary file1 (XLSX 2750 KB)

## Data Availability

The data set used for this meta-analysis is provided in Supplementary Table 1.

## References

[1] Handa T, Sasaki H, Takao M, Tano M, Uchida Y (2022) Proteomics-based investigation of cerebrovascular molecular mechanisms in cerebral amyloid angiopathy by the FFPE-LMD-PCT-SWATH method. Fluids Barriers CNS 19:56. 10.1186/s12987-022-00351-x 35778717 10.1186/s12987-022-00351-x PMC9250250

[2] Hondius DC, Eigenhuis KN, Morrema THJ, van der Schors RC, van Nierop P, Bugiani M et al (2018) Proteomics analysis identifies new markers associated with capillary cerebral amyloid angiopathy in Alzheimer’s disease. Acta Neuropathol Commun 6:46. 10.1186/s40478-018-0540-2 29860944 10.1186/s40478-018-0540-2 PMC5985582

[3] Inoue Y, Ueda M, Tasaki M, Takeshima A, Nagatoshi A, Masuda T et al (2017) Sushi repeat-containing protein 1: a novel disease-associated molecule in cerebral amyloid angiopathy. Acta Neuropathol 134:605–617. 10.1007/s00401-017-1720-z 28478503 10.1007/s00401-017-1720-z

[4] Leitner D, Kavanagh T, Kanshin E, Balcomb K, Pires G, Thierry M et al (2024) Differences in the cerebral amyloid angiopathy proteome in Alzheimer’s disease and mild cognitive impairment. Acta Neuropathol 148:9. 10.1007/s00401-024-02767-1 39039355 10.1007/s00401-024-02767-1 PMC11263258

[5] van Zalm PW, Ahmed S, Fatou B, Schreiber R, Barnaby O, Boxer A et al (2023) Meta-analysis of published cerebrospinal fluid proteomics data identifies and validates metabolic enzyme panel as Alzheimer’s disease biomarkers. Cell Rep Med 4:101005. 10.1016/j.xcrm.2023.101005 37075703 10.1016/j.xcrm.2023.101005 PMC10140596

[6] Yang AC, Vest RT, Kern F, Lee DP, Agam M, Maat CA et al (2022) A human brain vascular atlas reveals diverse mediators of Alzheimer’s risk. Nature 603:885–892. 10.1038/s41586-021-04369-3 35165441 10.1038/s41586-021-04369-3 PMC9635042

[7] Zellner A, Müller SA, Lindner B, Beaufort N, Rozemuller AJM, Arzberger T et al (2022) Proteomic profiling in cerebral amyloid angiopathy reveals an overlap with CADASIL highlighting accumulation of HTRA1 and its substrates. Acta Neuropathol Commun 10:6. 10.1186/s40478-021-01303-6 35074002 10.1186/s40478-021-01303-6 PMC8785498

